# A case of hypersomnia due to bilateral thalamic stroke

**DOI:** 10.1002/ccr3.1145

**Published:** 2017-08-24

**Authors:** Mohammed Osman, Ahmed Abdalla, Mohammed Al‐Qasmi, Ghassan Bachuwa

**Affiliations:** ^1^ Hurley Medical Center/Michigan State University One Hurley Plaza Flint 48503 Michigan

**Keywords:** Bilateral thalamic stroke, sleep‐like coma

## Abstract

Bilateral thalamic infarction (BTI) can present as sleep‐like coma without focal neurological signs which can lead to delay in the diagnosis. Due to the diagnostic challenge, treatment is often delayed. The early use of DWI‐MRI in suspected cases can help in the early diagnosis and treatment.

## Quiz Question

What is the diagnosis and what is the best imaging modality?

## Case

**Figure 1:**
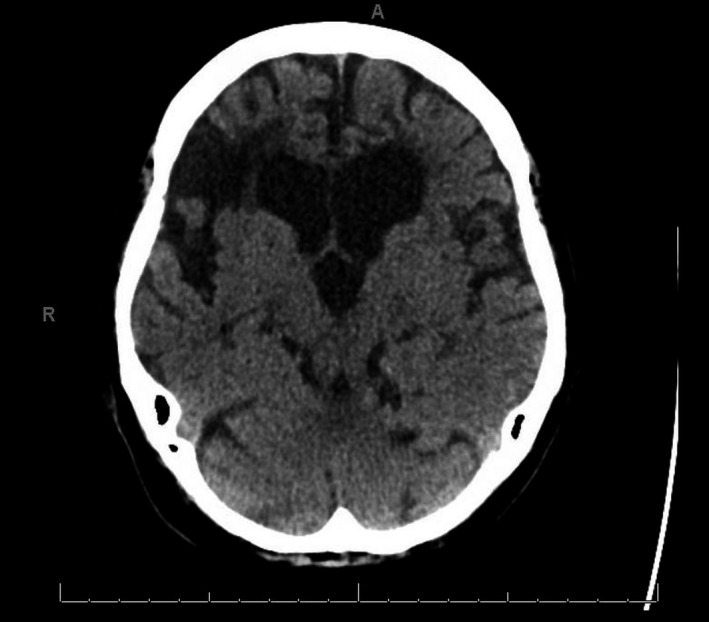
Computed tomography of the head showing bilateral old ischemic infarcts but no acute intracranial pathology.

**Figure 2:**
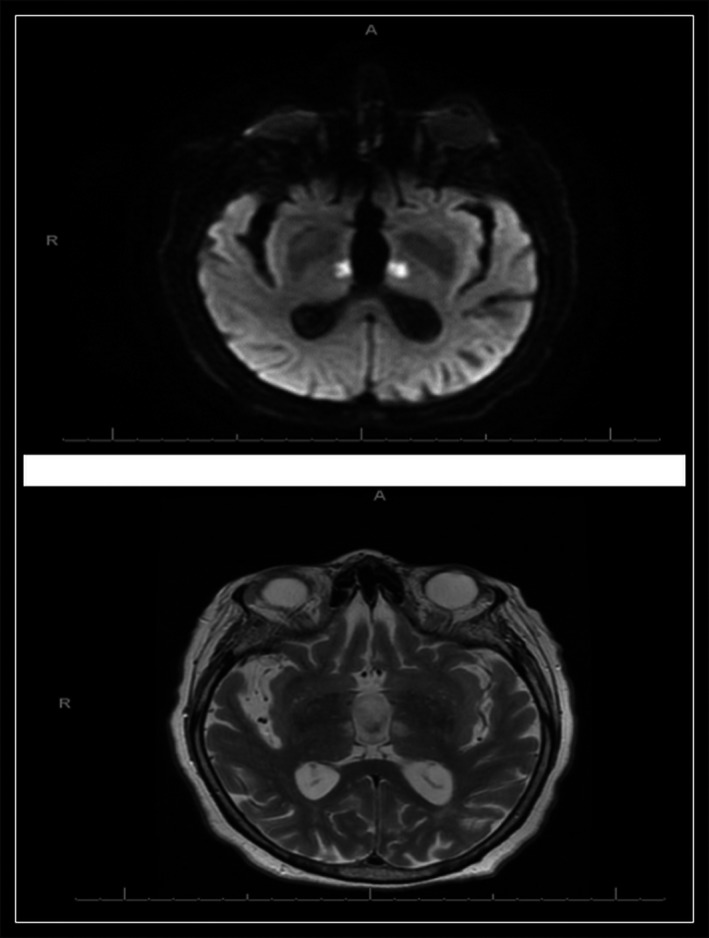
Diffusion‐weighted magnetic resonance imaging showing acute bilateral thalamic stroke.

Seventy eight‐year old female with past medical history of hypertension, type 2 diabetes mellitus, ischemic stroke with residual right‐side weakness, and paroxysmal atrial fibrillation. She presented to the hospital with altered mental status for 1 day. On examination, she was frequently yawning and sleeping and difficult to arouse. There were no new neurological deficits. Computed tomography of the head showed changes of multiple old infarcts (Fig. 1). Initial workup for infectious, toxic, and metabolic encephalopathy was unremarkable. A magnetic resonance imaging of the brain was performed and revealed acute symmetrical bilateral thalamic ischemic stroke (Fig 2).

The wide range sensorium impairment ranging from hypersomnia to coma secondary to bilateral thalamic infarction usually leads to diagnostic confusion among physicians 1. Many physicians might not consider stroke due to the lack of focal neurological deficit 1. In suspected cases, diagnosis can be made earlier with diffusion‐weighted (DW)‐MRI, this will lead to earlier intervention which in turn reduce the risk of deep coma due to involvement of the rostral midbrain and long‐term cognitive dysfunction including thalamic dementia 1, 2. Ironically, in a context in which time equals neuron, thrombolysis is rarely reported for thalamic infarction in the literature 1.

## Authorship

MO: planned, designed, and wrote the manuscript; prepared the images; had important role to facilitate the work between different authors; and also did the literature review. AA: planned, designed, wrote, and critically revised the manuscript; prepared the images; and also helped in the literature review. MA‐Q: planned, designed, and critically revised the manuscript; and played fundamental part in the image captions and selections of the appropriate images as well as in the discussion part. GB: planned designed wrote and critically revised the manuscript; and helped in writing learning points and key messages as well as in refining the final version.

## Conflict of Interest

None declared.
